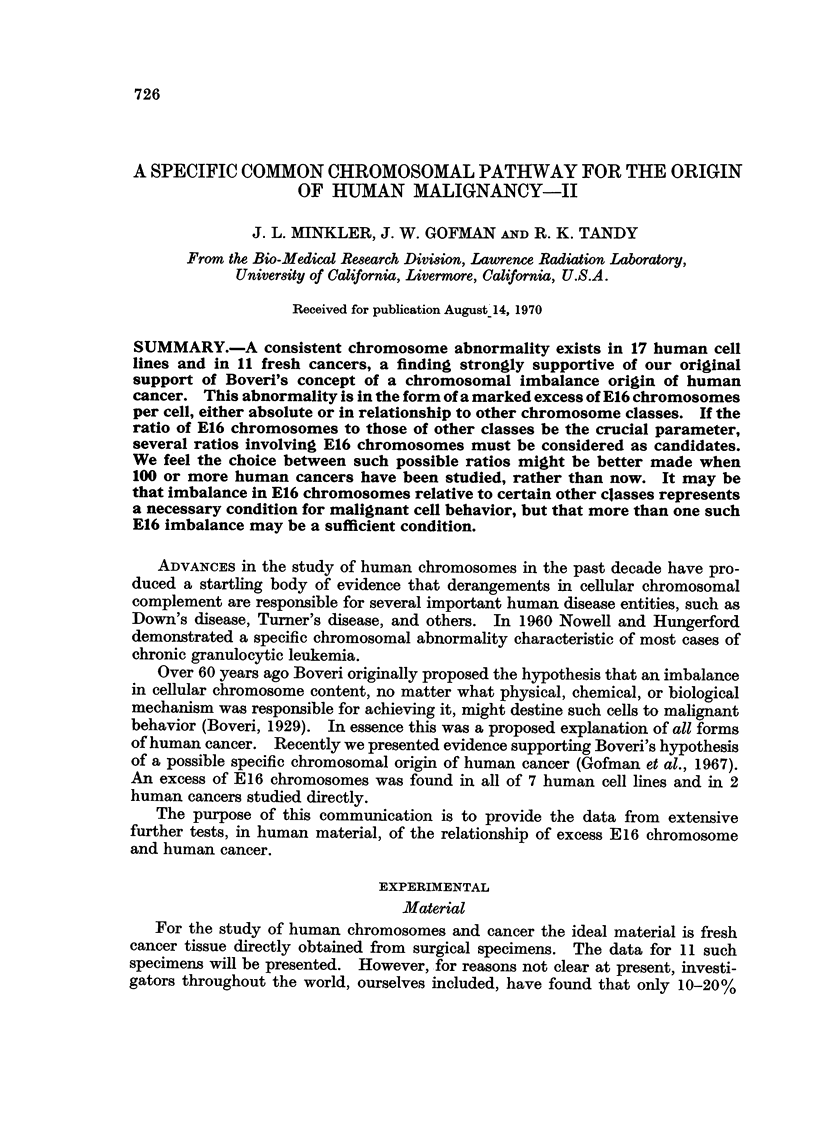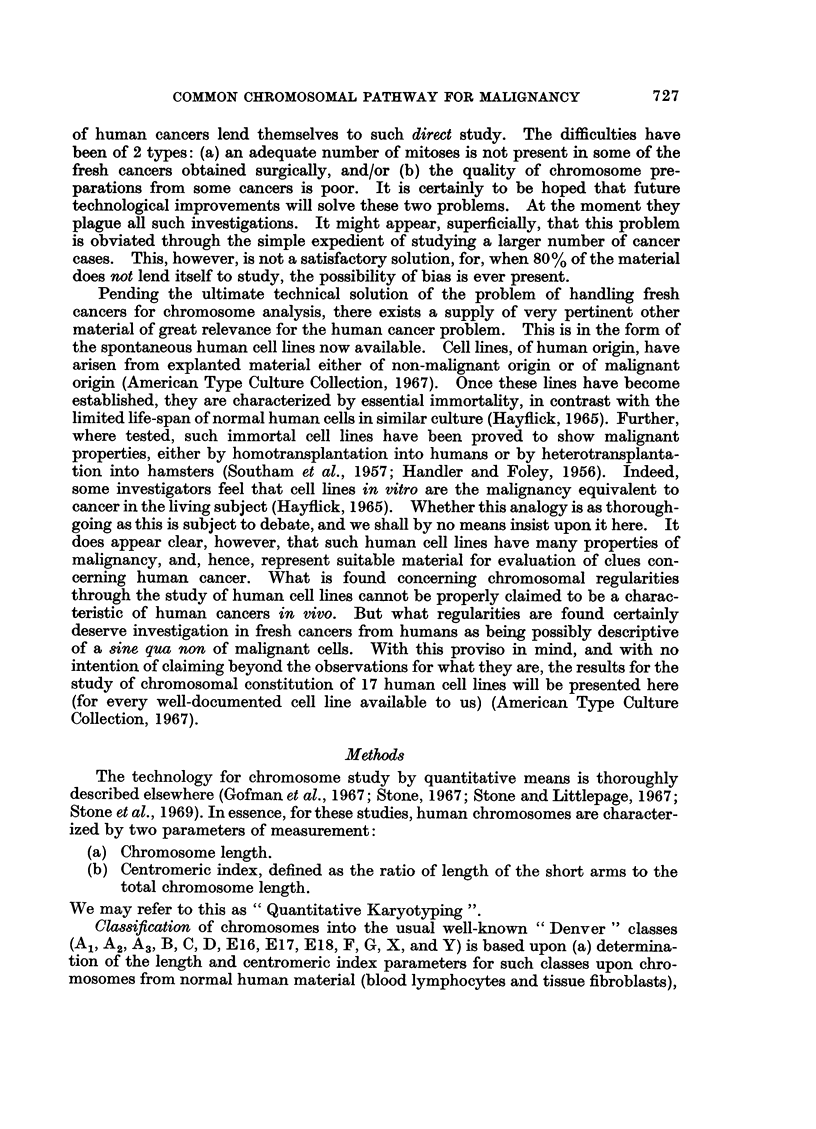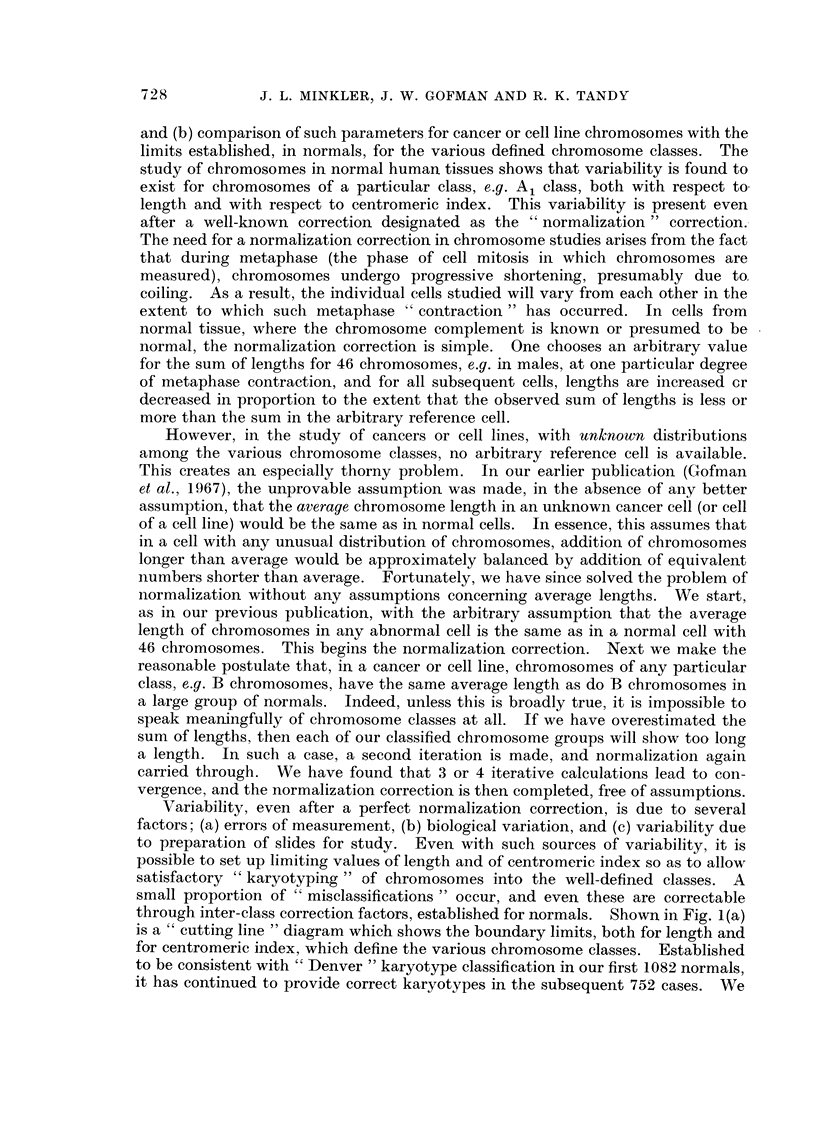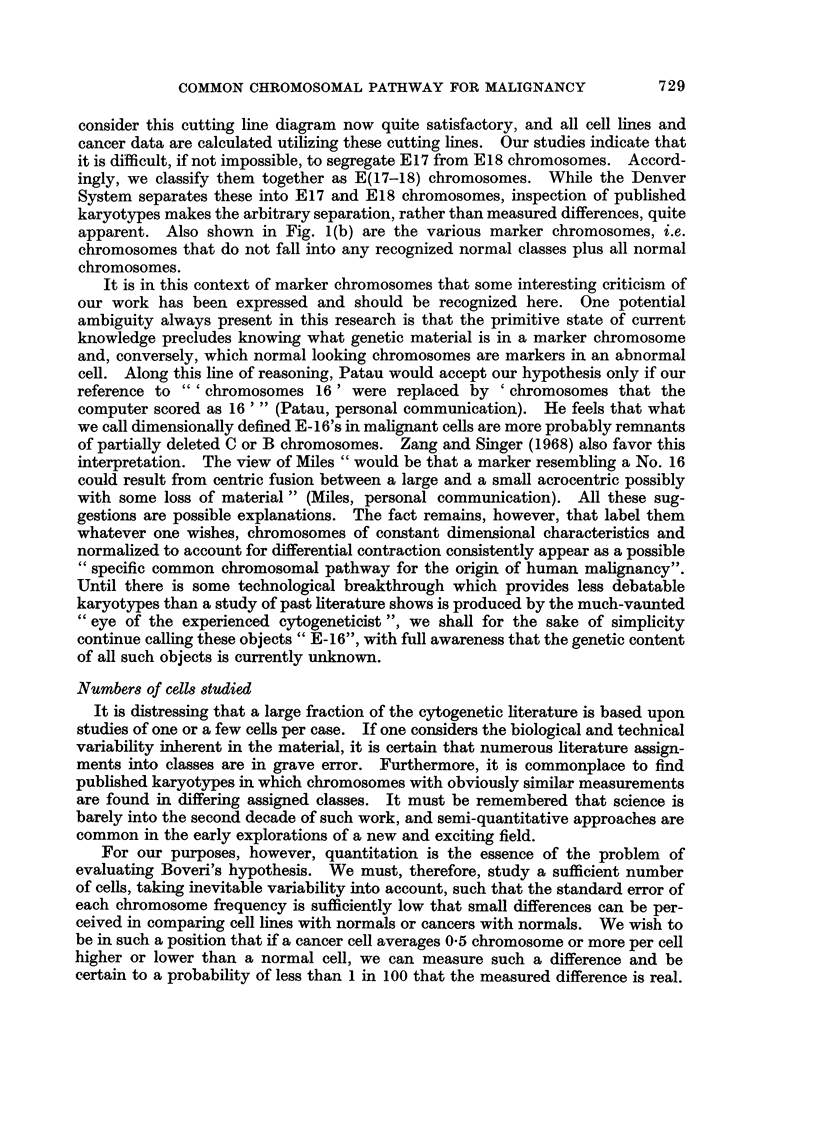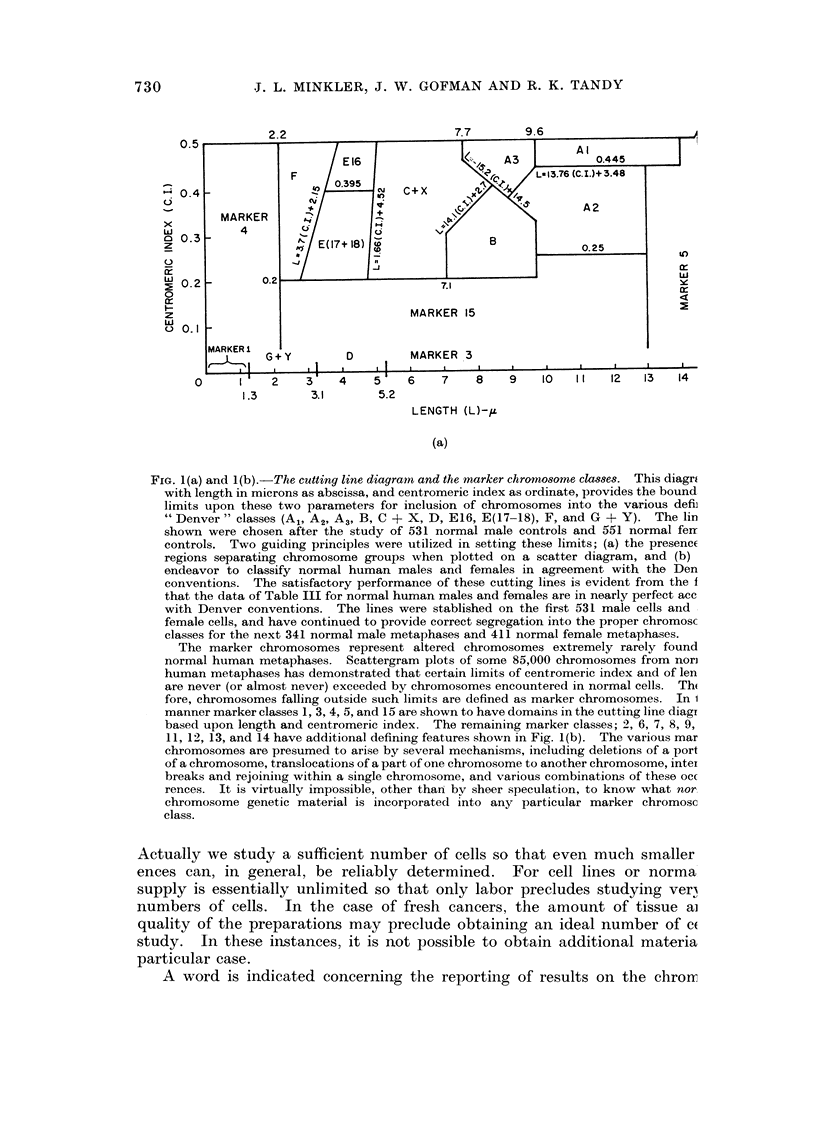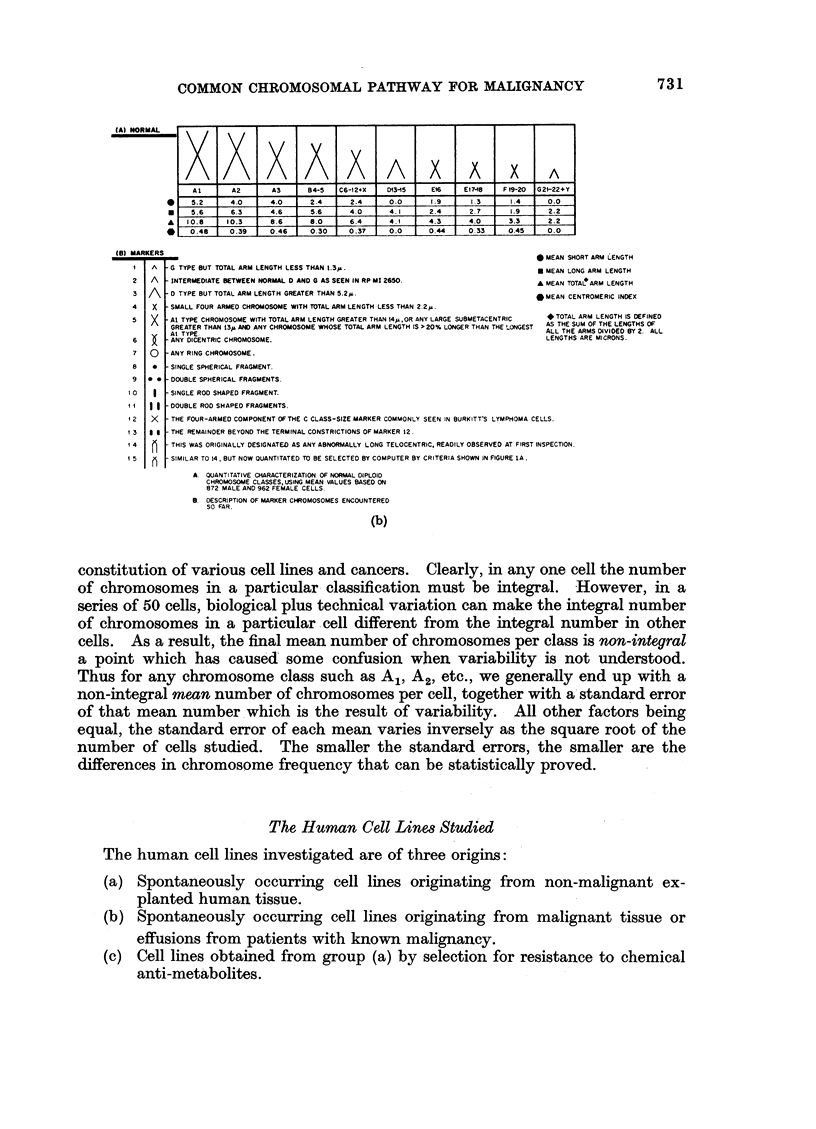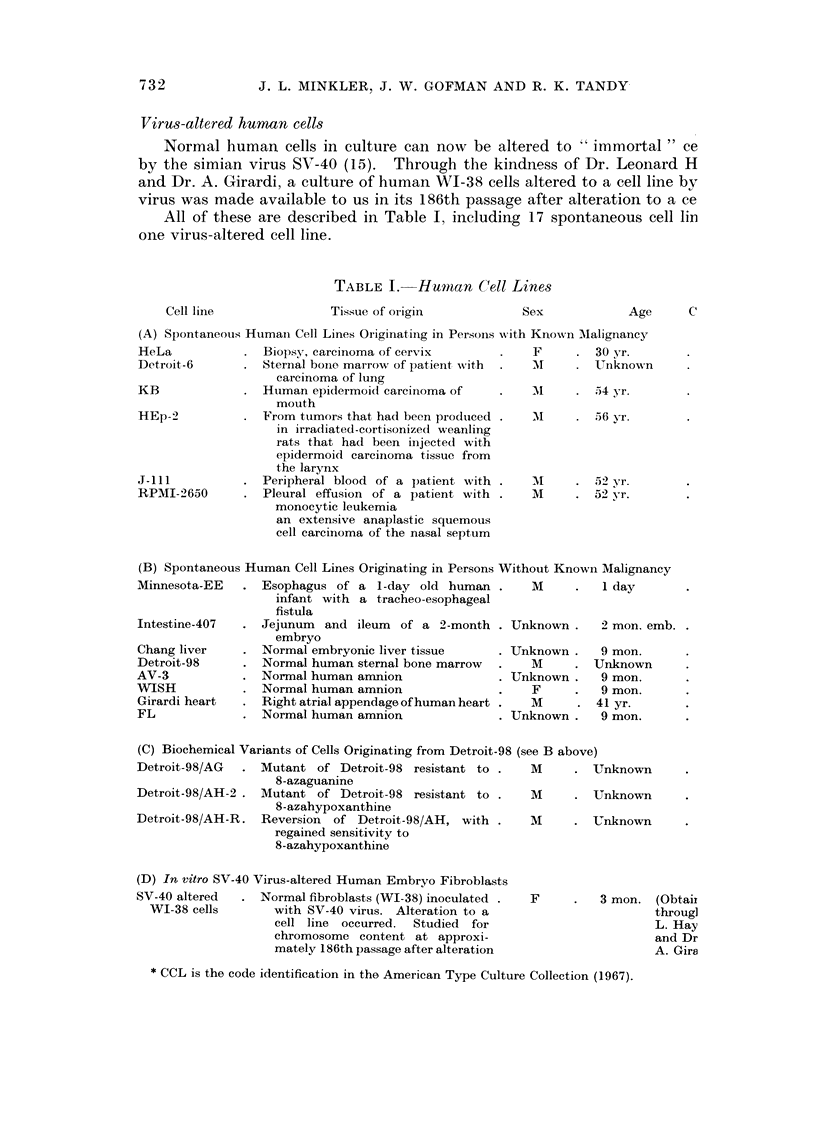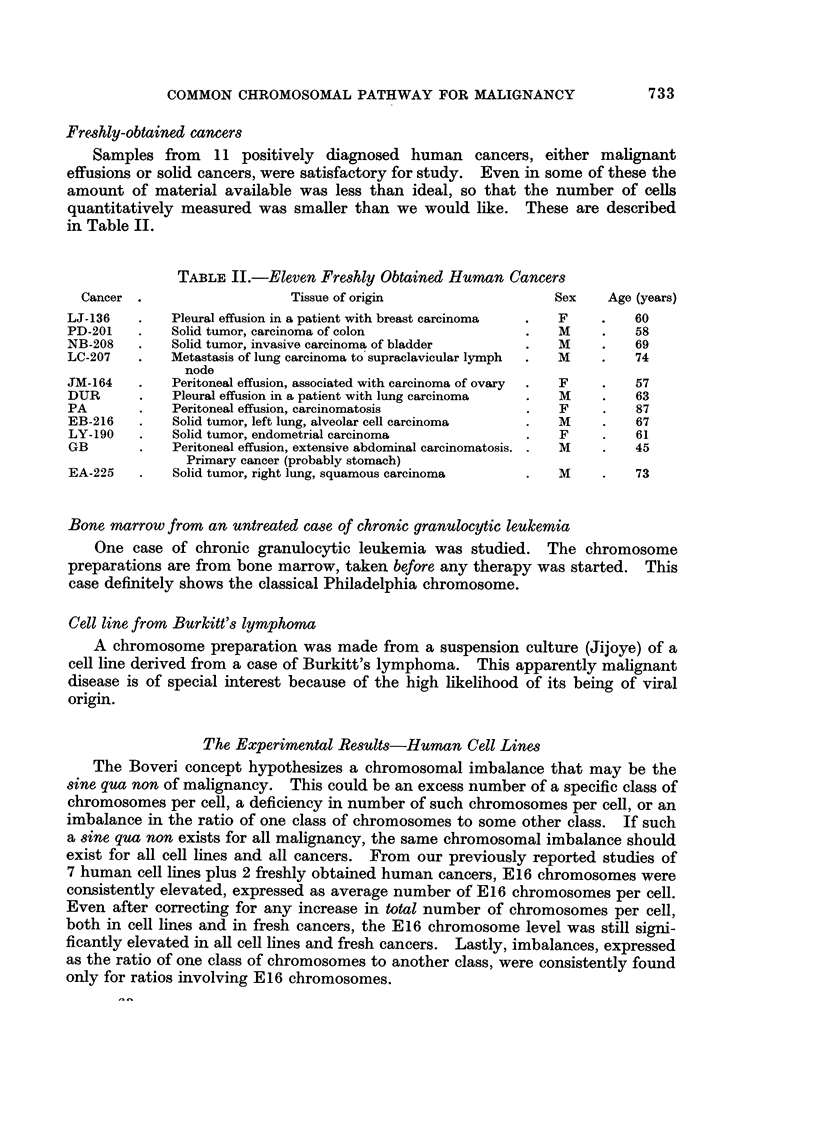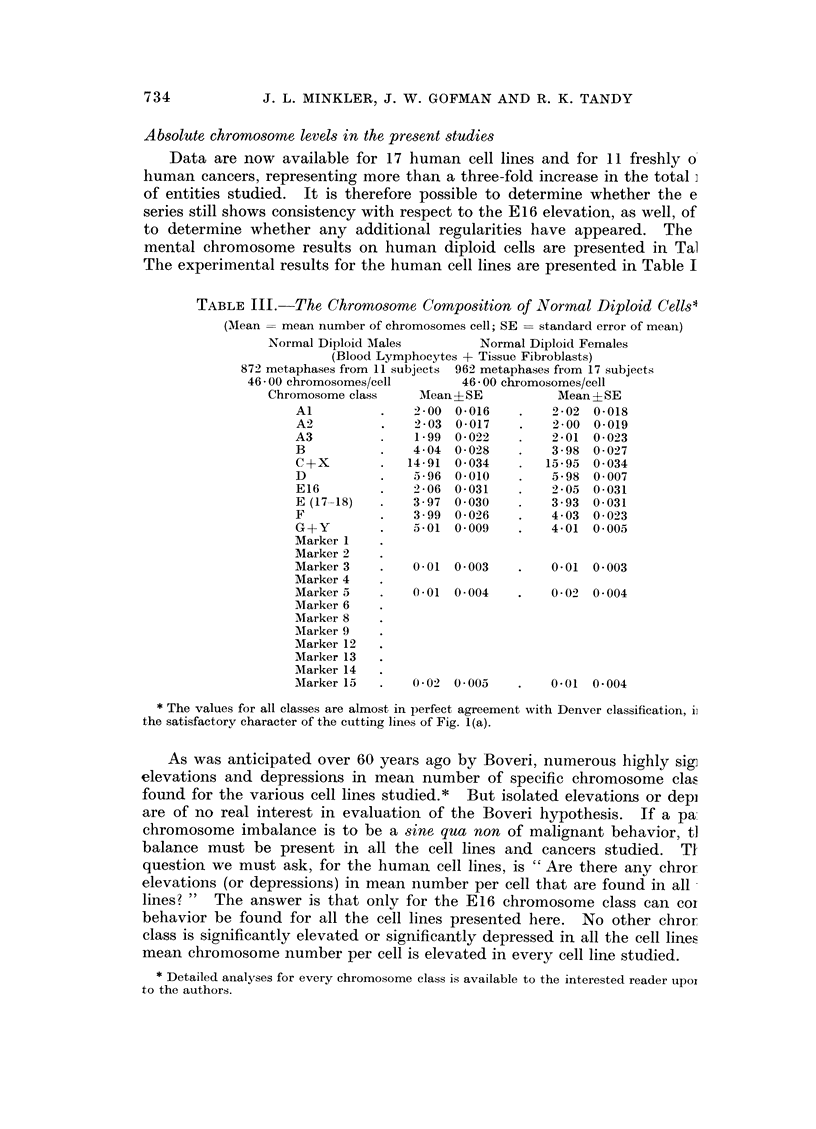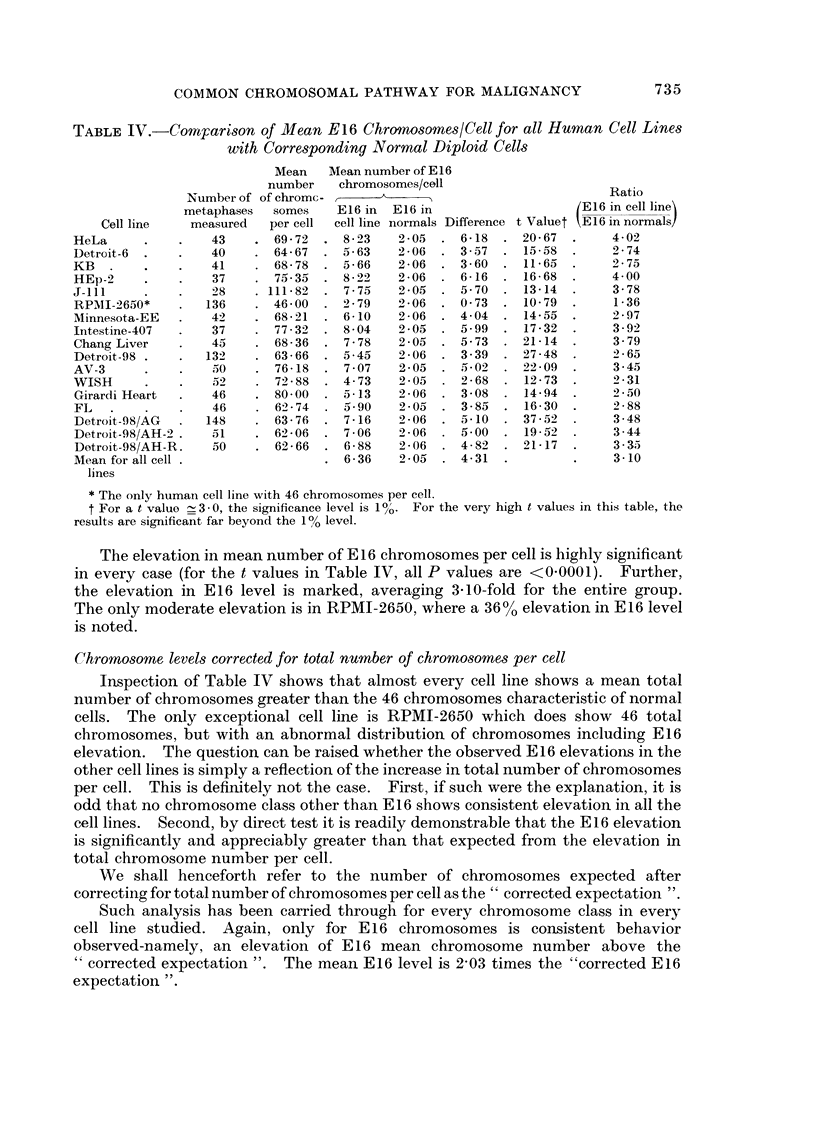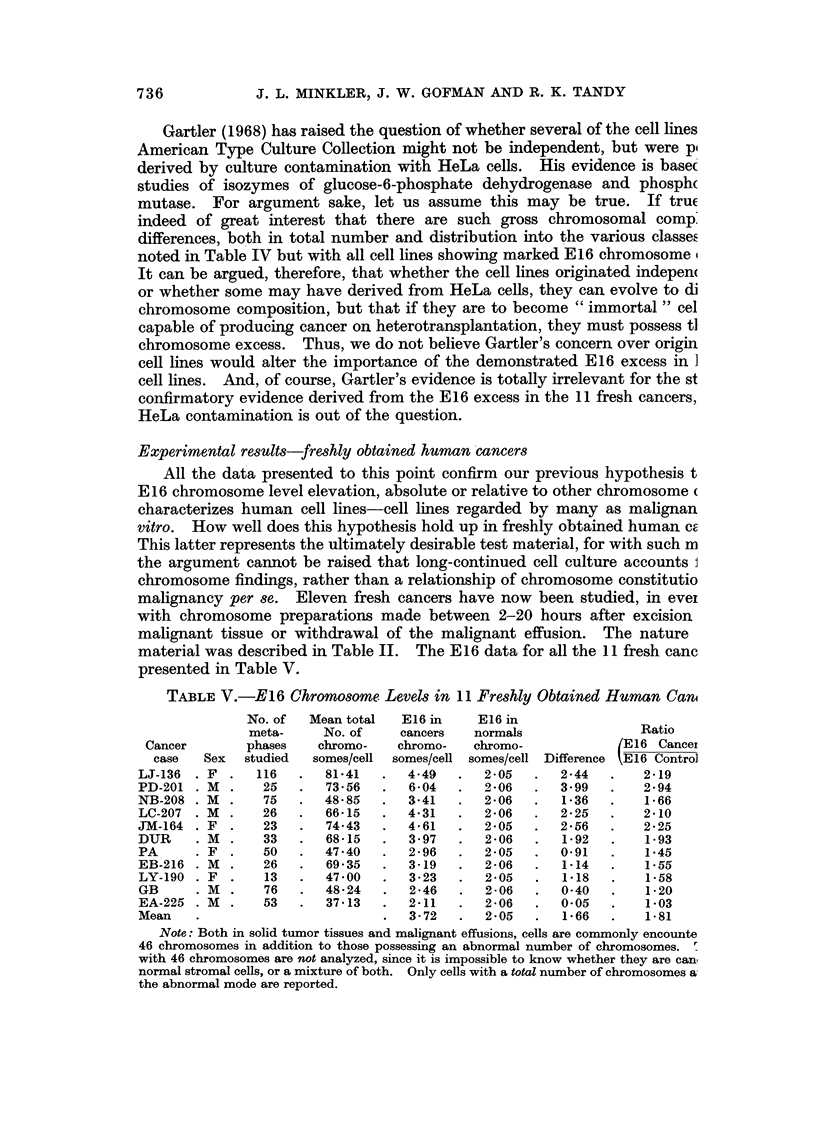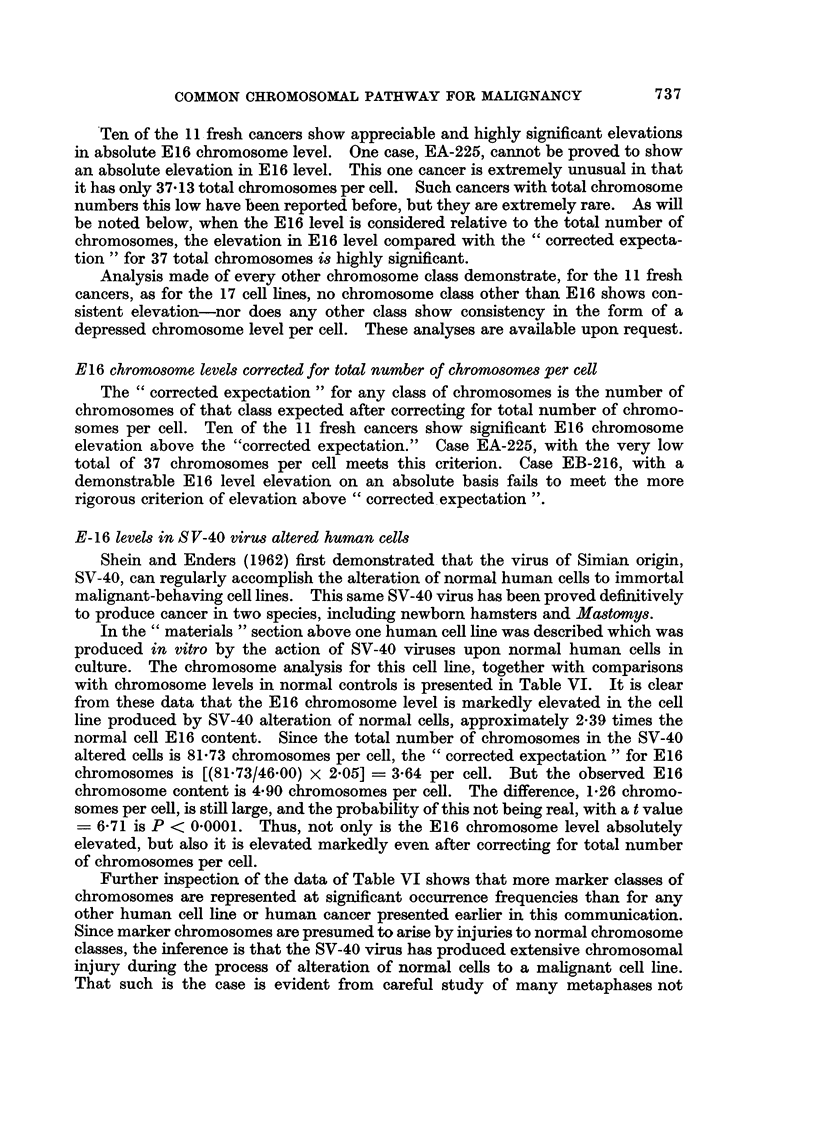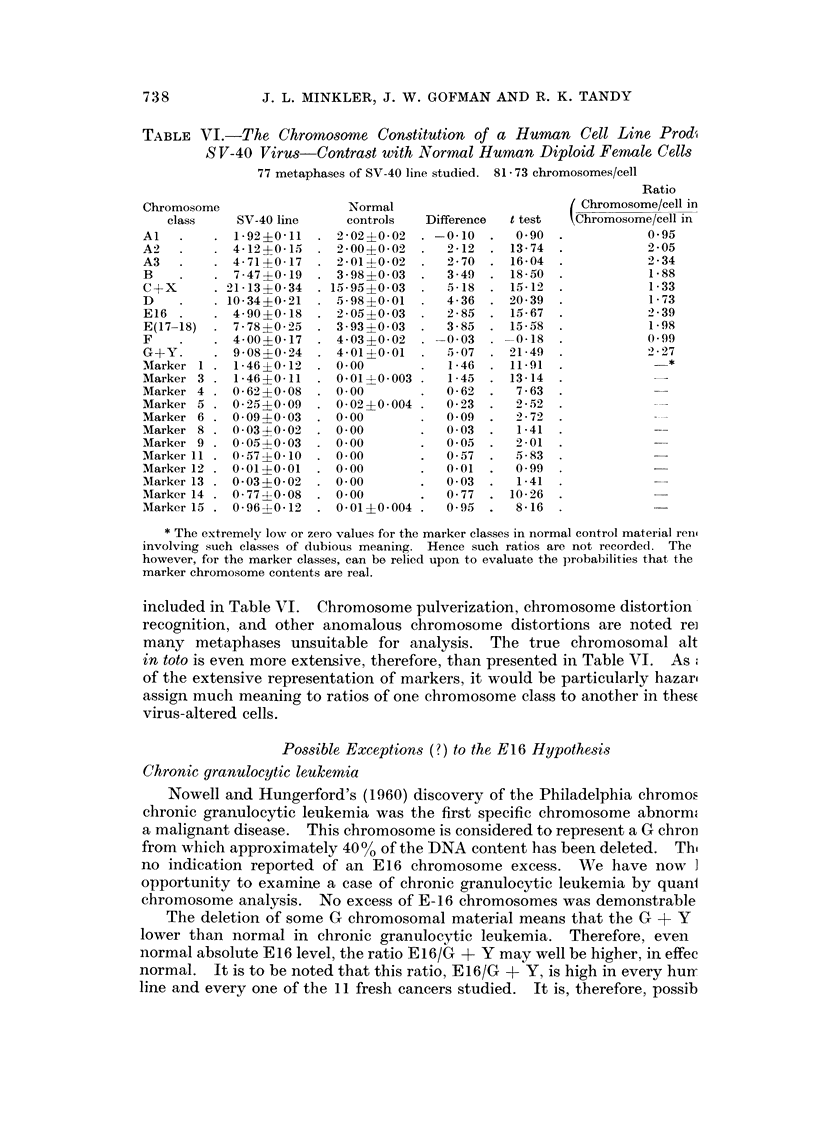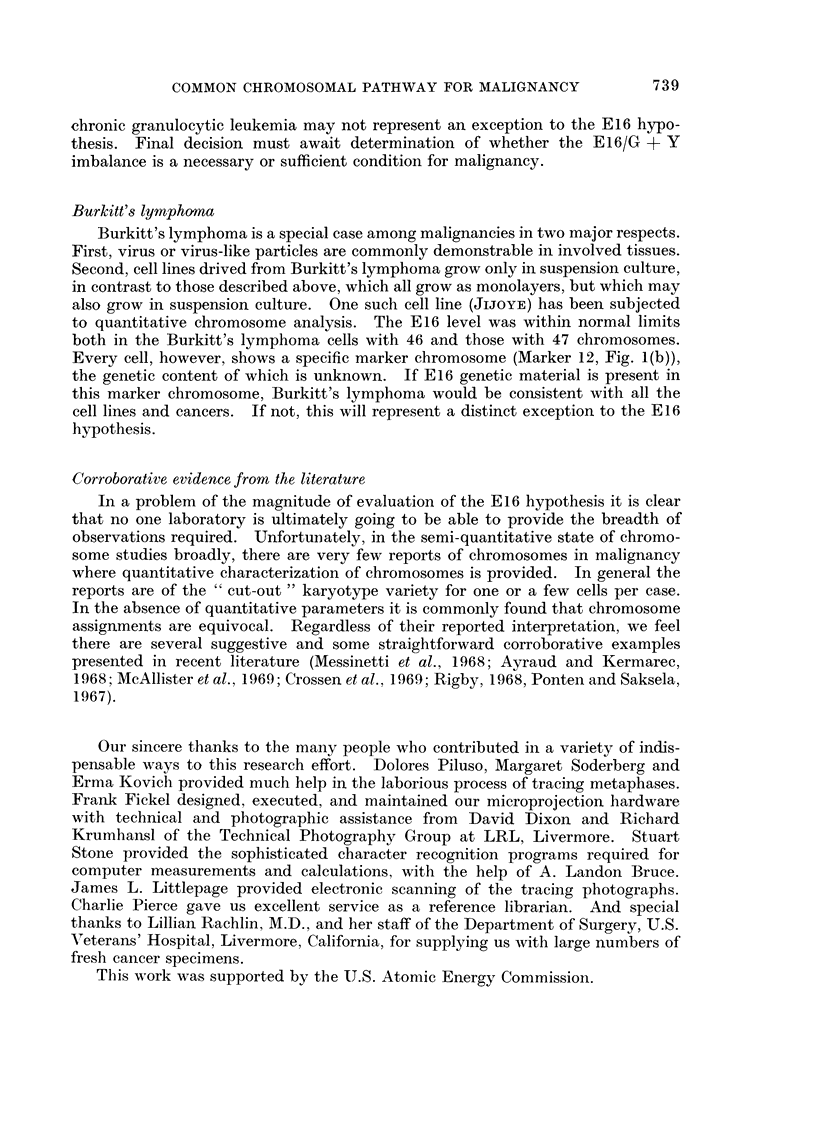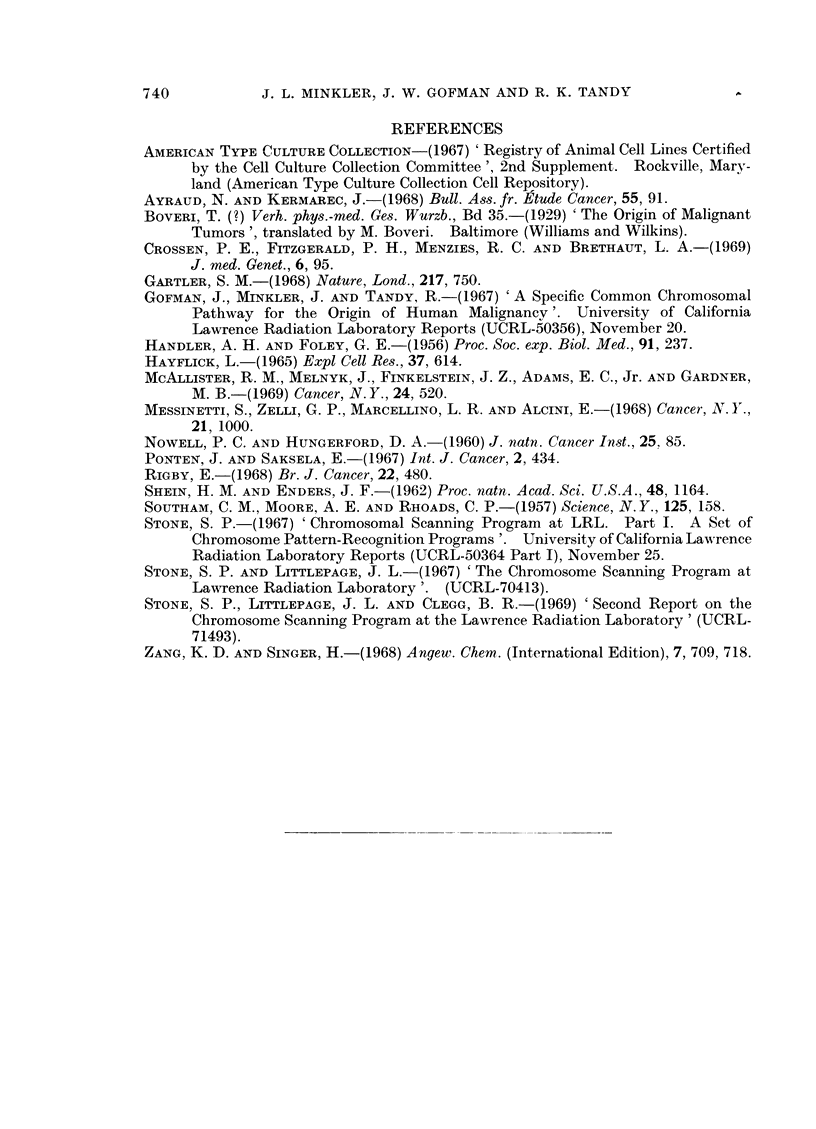# A Specific Common Chromosomal Pathway for the Origin of Human Malignancy—II

**DOI:** 10.1038/bjc.1970.87

**Published:** 1970-12

**Authors:** J. L. Minkler, J. W. Gofman, R. K. Tandy

## Abstract

A consistent chromosome abnormality exists in 17 human cell lines and in 11 fresh cancers, a finding strongly supportive of our original support of Boveri's concept of a chromosomal imbalance origin of human cancer. This abnormality is in the form of a marked excess of E16 chromosomes per cell, either absolute or in relationship to other chromosome classes. If the ratio of E16 chromosomes to those of other classes be the crucial parameter, several ratios involving E16 chromosomes must be considered as candidates. We feel the choice between such possible ratios might be better made when 100 or more human cancers have been studied, rather than now. It may be that imbalance in E16 chromosomes relative to certain other classes represents a necessary condition for malignant cell behavior, but that more than one such E16 imbalance may be a sufficient condition.


### A SPECIFIC COMMON CHROMOSOMAL PATHWAY FOR THE ORIGIN OF HUMAN MALIGNANCY—II

J. L. MINKLER, J. W. GOFMAN AND R. K. TANDY

*From the Bio-Medical Research Division, Lawrence Radiation Laboratory, University of California, Livermore, California, U.S.A.*

Received for publication August 14, 1970

**SUMMARY.—A consistent chromosome abnormality exists in 17 human cell lines and in 11 fresh cancers, a finding strongly supportive of our original support of Boveri's concept of a chromosomal imbalance origin of human cancer. This abnormality is in the form of a marked excess of E16 chromosomes per cell, either absolute or in relationship to other chromosome classes. If the ratio of E16 chromosomes to those of other classes be the crucial parameter, several ratios involving E16 chromosomes must be considered as candidates. We feel the choice between such possible ratios might be better made when 100 or more human cancers have been studied, rather than now. It may be that imbalance in E16 chromosomes relative to certain other classes represents a necessary condition for malignant cell behavior, but that more than one such E16 imbalance may be a sufficient condition.**

ADVANCES in the study of human chromosomes in the past decade have produced a startling body of evidence that derangements in cellular chromosomal complement are responsible for several important human disease entities, such as Down's disease, Turner's disease, and others. In 1960 Nowell and Hungerford demonstrated a specific chromosomal abnormality characteristic of most cases of chronic granulocytic leukemia.

Over 60 years ago Boveri originally proposed the hypothesis that an imbalance in cellular chromosome content, no matter what physical, chemical, or biological mechanism was responsible for achieving it, might destine such cells to malignant behavior (Boveri, 1929). In essence this was a proposed explanation of *all* forms of human cancer. Recently we presented evidence supporting Boveri's hypothesis of a possible specific chromosomal origin of human cancer (Gofman *et al.*, 1967). An excess of E16 chromosomes was found in all of 7 human cell lines and in 2 human cancers studied directly.

The purpose of this communication is to provide the data from extensive further tests, in human material, of the relationship of excess E16 chromosome and human cancer.

### EXPERIMENTAL

### *Material*

For the study of human chromosomes and cancer the ideal material is fresh cancer tissue directly obtained from surgical specimens. The data for 11 such specimens will be presented. However, for reasons not clear at present, investigators throughout the world, ourselves included, have found that only 10–20%

of human cancers lend themselves to such *direct* study. The difficulties have been of 2 types: (a) an adequate number of mitoses is not present in some of the fresh cancers obtained surgically, and/or (b) the quality of chromosome preparations from some cancers is poor. It is certainly to be hoped that future technological improvements will solve these two problems. At the moment they plague all such investigations. It might appear, superficially, that this problem is obviated through the simple expedient of studying a larger number of cancer cases. This, however, is not a satisfactory solution, for, when 80% of the material does *not* lend itself to study, the possibility of bias is ever present.

Pending the ultimate technical solution of the problem of handling fresh cancers for chromosome analysis, there exists a supply of very pertinent other material of great relevance for the human cancer problem. This is in the form of the spontaneous human cell lines now available. Cell lines, of human origin, have arisen from explanted material either of non-malignant origin or of malignant origin (American Type Culture Collection, 1967). Once these lines have become established, they are characterized by essential immortality, in contrast with the limited life-span of normal human cells in similar culture (Hayflick, 1965). Further, where tested, such immortal cell lines have been proved to show malignant properties, either by homotransplantation into humans or by heterotransplantation into hamsters (Southam *et al.*, 1957; Handler and Foley, 1956). Indeed, some investigators feel that cell lines *in vitro* are the malignancy equivalent to cancer in the living subject (Hayflick, 1965). Whether this analogy is as thoroughgoing as this is subject to debate, and we shall by no means insist upon it here. It does appear clear, however, that such human cell lines have many properties of malignancy, and, hence, represent suitable material for evaluation of clues concerning human cancer. What is found concerning chromosomal regularities through the study of human cell lines cannot be properly claimed to be a characteristic of human cancers *in vivo*. But what regularities are found certainly deserve investigation in fresh cancers from humans as being possibly descriptive of a *sine qua non* of malignant cells. With this proviso in mind, and with no intention of claiming beyond the observations for what they are, the results for the study of chromosomal constitution of 17 human cell lines will be presented here (for every well-documented cell line available to us) (American Type Culture Collection, 1967).

### *Methods*

The technology for chromosome study by quantitative means is thoroughly described elsewhere (Gofman *et al.*, 1967; Stone, 1967; Stone and Littlepage, 1967; Stone *et al.*, 1969). In essence, for these studies, human chromosomes are characterized by two parameters of measurement:

(a) Chromosome length.
(b) Centromeric index, defined as the ratio of length of the short arms to the total chromosome length.

We may refer to this as " Quantitative Karyotyping ".

*Classification* of chromosomes into the usual well-known " Denver " classes ($A_1$, $A_2$, $A_3$, B, C, D, E16, E17, E18, F, G, X, and Y) is based upon (a) determination of the length and centromeric index parameters for such classes upon chromosomes from normal human material (blood lymphocytes and tissue fibroblasts), and (b) comparison of such parameters for cancer or cell line chromosomes with the limits established, in normals, for the various defined chromosome classes. The study of chromosomes in normal human tissues shows that variability is found to exist for chromosomes of a particular class, *e.g.* $A_1$ class, both with respect to length and with respect to centromeric index. This variability is present even after a well-known correction designated as the " normalization " correction. The need for a normalization correction in chromosome studies arises from the fact that during metaphase (the phase of cell mitosis in which chromosomes are measured), chromosomes undergo progressive shortening, presumably due to coiling. As a result, the individual cells studied will vary from each other in the extent to which such metaphase " contraction " has occurred. In cells from normal tissue, where the chromosome complement is known or presumed to be normal, the normalization correction is simple. One chooses an arbitrary value for the sum of lengths for 46 chromosomes, *e.g.* in males, at one particular degree of metaphase contraction, and for all subsequent cells, lengths are increased or decreased in proportion to the extent that the observed sum of lengths is less or more than the sum in the arbitrary reference cell.

However, in the study of cancers or cell lines, with *unknown* distributions among the various chromosome classes, no arbitrary reference cell is available. This creates an especially thorny problem. In our earlier publication (Gofman *et al.*, 1967), the unprovable assumption was made, in the absence of any better assumption, that the *average* chromosome length in an unknown cancer cell (or cell of a cell line) would be the same as in normal cells. In essence, this assumes that in a cell with any unusual distribution of chromosomes, addition of chromosomes longer than average would be approximately balanced by addition of equivalent numbers shorter than average. Fortunately, we have since solved the problem of normalization without any assumptions concerning average lengths. We start, as in our previous publication, with the arbitrary assumption that the average length of chromosomes in any abnormal cell is the same as in a normal cell with 46 chromosomes. This begins the normalization correction. Next we make the reasonable postulate that, in a cancer or cell line, chromosomes of any particular class, *e.g.* B chromosomes, have the same average length as do B chromosomes in a large group of normals. Indeed, unless this is broadly true, it is impossible to speak meaningfully of chromosome classes at all. If we have overestimated the sum of lengths, then each of our classified chromosome groups will show too long a length. In such a case, a second iteration is made, and normalization again carried through. We have found that 3 or 4 iterative calculations lead to convergence, and the normalization correction is then completed, free of assumptions.

Variability, even after a perfect normalization correction, is due to several factors; (a) errors of measurement, (b) biological variation, and (c) variability due to preparation of slides for study. Even with such sources of variability, it is possible to set up limiting values of length and of centromeric index so as to allow satisfactory " karyotyping " of chromosomes into the well-defined classes. A small proportion of " misclassifications " occur, and even these are correctable through inter-class correction factors, established for normals. Shown in Fig. 1(a) is a " cutting line " diagram which shows the boundary limits, both for length and for centromeric index, which define the various chromosome classes. Established to be consistent with " Denver " karyotype classification in our first 1082 normals, it has continued to provide correct karyotypes in the subsequent 752 cases. We consider this cutting line diagram now quite satisfactory, and all cell lines and cancer data are calculated utilizing these cutting lines. Our studies indicate that it is difficult, if not impossible, to segregate E17 from E18 chromosomes. Accordingly, we classify them together as E(17–18) chromosomes. While the Denver System separates these into E17 and E18 chromosomes, inspection of published karyotypes makes the arbitrary separation, rather than measured differences, quite apparent. Also shown in Fig. 1(b) are the various marker chromosomes, *i.e.* chromosomes that do not fall into any recognized normal classes plus all normal chromosomes.

It is in this context of marker chromosomes that some interesting criticism of our work has been expressed and should be recognized here. One potential ambiguity always present in this research is that the primitive state of current knowledge precludes knowing what genetic material is in a marker chromosome and, conversely, which normal looking chromosomes are markers in an abnormal cell. Along this line of reasoning, Patau would accept our hypothesis only if our reference to " ' chromosomes 16 ' were replaced by ' chromosomes that the computer scored as 16 ' " (Patau, personal communication). He feels that what we call dimensionally defined E-16's in malignant cells are more probably remnants of partially deleted C or B chromosomes. Zang and Singer (1968) also favor this interpretation. The view of Miles " would be that a marker resembling a No. 16 could result from centric fusion between a large and a small acrocentric possibly with some loss of material " (Miles, personal communication). All these suggestions are possible explanations. The fact remains, however, that label them whatever one wishes, chromosomes of constant dimensional characteristics and normalized to account for differential contraction consistently appear as a possible " specific common chromosomal pathway for the origin of human malignancy". Until there is some technological breakthrough which provides less debatable karyotypes than a study of past literature shows is produced by the much-vaunted " eye of the experienced cytogeneticist ", we shall for the sake of simplicity continue calling these objects " E-16", with full awareness that the genetic content of all such objects is currently unknown.

*Numbers of cells studied*

It is distressing that a large fraction of the cytogenetic literature is based upon studies of one or a few cells per case. If one considers the biological and technical variability inherent in the material, it is certain that numerous literature assignments into classes are in grave error. Furthermore, it is commonplace to find published karyotypes in which chromosomes with obviously similar measurements are found in differing assigned classes. It must be remembered that science is barely into the second decade of such work, and semi-quantitative approaches are common in the early explorations of a new and exciting field.

For our purposes, however, quantitation is the essence of the problem of evaluating Boveri's hypothesis. We must, therefore, study a sufficient number of cells, taking inevitable variability into account, such that the standard error of each chromosome frequency is sufficiently low that small differences can be perceived in comparing cell lines with normals or cancers with normals. We wish to be in such a position that if a cancer cell averages 0·5 chromosome or more per cell higher or lower than a normal cell, we can measure such a difference and be certain to a probability of less than 1 in 100 that the measured difference is real.

(a)

FIG. 1(a) and 1(b).—*The cutting line diagram and the marker chromosome classes.* This diagr with length in microns as abscissa, and centromeric index as ordinate, provides the bound limits upon these two parameters for inclusion of chromosomes into the various defi "Denver" classes ($A_1$, $A_2$, $A_3$, B, C + X, D, E16, E(17–18), F, and G + Y). The lin shown were chosen after the study of 531 normal male controls and 551 normal fen controls. Two guiding principles were utilized in setting these limits; (a) the presence regions separating chromosome groups when plotted on a scatter diagram, and (b) endeavor to classify normal human males and females in agreement with the Den conventions. The satisfactory performance of these cutting lines is evident from the f that the data of Table III for normal human males and females are in nearly perfect acc with Denver conventions. The lines were stablished on the first 531 male cells and female cells, and have continued to provide correct segregation into the proper chromosc classes for the next 341 normal male metaphases and 411 normal female metaphases.

The marker chromosomes represent altered chromosomes extremely rarely found normal human metaphases. Scattergram plots of some 85,000 chromosomes from nor human metaphases has demonstrated that certain limits of centromeric index and of len are never (or almost never) exceeded by chromosomes encountered in normal cells. The fore, chromosomes falling outside such limits are defined as marker chromosomes. In t manner marker classes 1, 3, 4, 5, and 15 are shown to have domains in the cutting line diagr based upon length and centromeric index. The remaining marker classes; 2, 6, 7, 8, 9, 11, 12, 13, and 14 have additional defining features shown in Fig. 1(b). The various mar chromosomes are presumed to arise by several mechanisms, including deletions of a port of a chromosome, translocations of a part of one chromosome to another chromosome, inte breaks and rejoining within a single chromosome, and various combinations of these occ rences. It is virtually impossible, other than by sheer speculation, to know what *nor* chromosome genetic material is incorporated into any particular marker chromoso class.

Actually we study a sufficient number of cells so that even much smaller ences can, in general, be reliably determined. For cell lines or norma supply is essentially unlimited so that only labor precludes studying ver numbers of cells. In the case of fresh cancers, the amount of tissue a quality of the preparations may preclude obtaining an ideal number of c study. In these instances, it is not possible to obtain additional materia particular case.

A word is indicated concerning the reporting of results on the chrom

(A) NORMAL

| | A1 | A2 | A3 | B4-5 | C6-12+X | D13-15 | E16 | E17-18 | F19-20 | G21-22+Y |
|---|---|---|---|---|---|---|---|---|---|---|
| Mean short arm length | 5.2 | 4.0 | 4.0 | 2.4 | 2.4 | 0.0 | 1.9 | 1.3 | 1.4 | 0.0 |
| Mean long arm length | 5.6 | 6.3 | 4.6 | 5.6 | 4.0 | 4.1 | 2.4 | 2.7 | 1.9 | 2.2 |
| Mean total arm length | 10.8 | 10.3 | 8.6 | 8.0 | 6.4 | 4.1 | 4.3 | 4.0 | 3.3 | 2.2 |
| Mean centromeric index | 0.48 | 0.39 | 0.46 | 0.30 | 0.37 | 0.0 | 0.44 | 0.33 | 0.45 | 0.0 |

(B) MARKERS

1 G TYPE BUT TOTAL ARM LENGTH LESS THAN 1.3μ.

2 INTERMEDIATE BETWEEN NORMAL D AND G AS SEEN IN RP MI 2650.

3 D TYPE BUT TOTAL ARM LENGTH GREATER THAN 5.2μ.

4 SMALL FOUR ARMED CHROMOSOME WITH TOTAL ARM LENGTH LESS THAN 2.2μ.

5 A1 TYPE CHROMOSOME WITH TOTAL ARM LENGTH GREATER THAN 14μ, OR ANY LARGE SUBMETACENTRIC GREATER THAN 13μ AND ANY CHROMOSOME WHOSE TOTAL ARM LENGTH IS > 20% LONGER THAN THE LONGEST A1 TYPE.

6 ANY DICENTRIC CHROMOSOME.

7 ANY RING CHROMOSOME.

8 SINGLE SPHERICAL FRAGMENT.

9 DOUBLE SPHERICAL FRAGMENTS.

10 SINGLE ROD SHAPED FRAGMENT.

11 DOUBLE ROD SHAPED FRAGMENTS.

12 THE FOUR-ARMED COMPONENT OF THE C CLASS-SIZE MARKER COMMONLY SEEN IN BURKITT'S LYMPHOMA CELLS.

13 THE REMAINDER BEYOND THE TERMINAL CONSTRICTIONS OF MARKER 12.

14 THIS WAS ORIGINALLY DESIGNATED AS ANY ABNORMALLY LONG TELOCENTRIC, READILY OBSERVED AT FIRST INSPECTION.

15 SIMILAR TO 14, BUT NOW QUANTITATED TO BE SELECTED BY COMPUTER BY CRITERIA SHOWN IN FIGURE 1A.

● MEAN SHORT ARM LENGTH

■ MEAN LONG ARM LENGTH

▲ MEAN TOTAL◆ ARM LENGTH

● MEAN CENTROMERIC INDEX

◆ TOTAL ARM LENGTH IS DEFINED AS THE SUM OF THE LENGTHS OF ALL THE ARMS DIVIDED BY 2. ALL LENGTHS ARE MICRONS.

A. QUANTITATIVE CHARACTERIZATION OF NORMAL DIPLOID CHROMOSOME CLASSES, USING MEAN VALUES BASED ON 872 MALE AND 962 FEMALE CELLS.

B. DESCRIPTION OF MARKER CHROMOSOMES ENCOUNTERED SO FAR.

(b)

constitution of various cell lines and cancers. Clearly, in any one cell the number of chromosomes in a particular classification must be integral. However, in a series of 50 cells, biological plus technical variation can make the integral number of chromosomes in a particular cell different from the integral number in other cells. As a result, the final mean number of chromosomes per class is *non-integral* a point which has caused some confusion when variability is not understood. Thus for any chromosome class such as $A_1$, $A_2$, etc., we generally end up with a non-integral *mean* number of chromosomes per cell, together with a standard error of that mean number which is the result of variability. All other factors being equal, the standard error of each mean varies inversely as the square root of the number of cells studied. The smaller the standard errors, the smaller are the differences in chromosome frequency that can be statistically proved.

### *The Human Cell Lines Studied*

The human cell lines investigated are of three origins:

(a) Spontaneously occurring cell lines originating from non-malignant explanted human tissue.
(b) Spontaneously occurring cell lines originating from malignant tissue or effusions from patients with known malignancy.
(c) Cell lines obtained from group (a) by selection for resistance to chemical anti-metabolites.

*Virus-altered human cells*

Normal human cells in culture can now be altered to " immortal " ce by the simian virus SV-40 (15). Through the kindness of Dr. Leonard H and Dr. A. Girardi, a culture of human WI-38 cells altered to a cell line by virus was made available to us in its 186th passage after alteration to a ce

All of these are described in Table I, including 17 spontaneous cell lin one virus-altered cell line.

TABLE I.—*Human Cell Lines*

| Cell line | Tissue of origin | Sex | Age | C |
|---|---|---|---|---|
| (A) Spontaneous Human Cell Lines Originating in Persons with Known Malignancy | | | | |
| HeLa | Biopsy, carcinoma of cervix | F | 30 yr. | . |
| Detroit-6 | Sternal bone marrow of patient with carcinoma of lung | M | Unknown | . |
| KB | Human epidermoid carcinoma of mouth | M | 54 yr. | . |
| HEp-2 | From tumors that had been produced in irradiated-cortisonized weanling rats that had been injected with epidermoid carcinoma tissue from the larynx | M | 56 yr. | . |
| J-111 | Peripheral blood of a patient with monocytic leukemia | M | 52 yr. | . |
| RPMI-2650 | Pleural effusion of a patient with an extensive anaplastic squemous cell carcinoma of the nasal septum | M | 52 yr. | . |
| (B) Spontaneous Human Cell Lines Originating in Persons Without Known Malignancy | | | | |
| Minnesota-EE | Esophagus of a 1-day old human infant with a tracheo-esophageal fistula | M | 1 day | . |
| Intestine-407 | Jejunum and ileum of a 2-month embryo | Unknown | 2 mon. emb. | . |
| Chang liver | Normal embryonic liver tissue | Unknown | 9 mon. | . |
| Detroit-98 | Normal human sternal bone marrow | M | Unknown | . |
| AV-3 | Normal human amnion | Unknown | 9 mon. | . |
| WISH | Normal human amnion | F | 9 mon. | . |
| Girardi heart | Right atrial appendage of human heart | M | 41 yr. | . |
| FL | Normal human amnion | Unknown | 9 mon. | . |
| (C) Biochemical Variants of Cells Originating from Detroit-98 (see B above) | | | | |
| Detroit-98/AG | Mutant of Detroit-98 resistant to 8-azaguanine | M | Unknown | . |
| Detroit-98/AH-2 | Mutant of Detroit-98 resistant to 8-azahypoxanthine | M | Unknown | . |
| Detroit-98/AH-R | Reversion of Detroit-98/AH, with regained sensitivity to 8-azahypoxanthine | M | Unknown | . |
| (D) *In vitro* SV-40 Virus-altered Human Embryo Fibroblasts | | | | |
| SV-40 altered WI-38 cells | Normal fibroblasts (WI-38) inoculated with SV-40 virus. Alteration to a cell line occurred. Studied for chromosome content at approximately 186th passage after alteration | F | 3 mon. | (Obtai throug L. Hay and Dr A. Gira |

* CCL is the code identification in the American Type Culture Collection (1967).

*Freshly-obtained cancers*

Samples from 11 positively diagnosed human cancers, either malignant effusions or solid cancers, were satisfactory for study. Even in some of these the amount of material available was less than ideal, so that the number of cells quantitatively measured was smaller than we would like. These are described in Table II.

TABLE II.—*Eleven Freshly Obtained Human Cancers*

| Cancer | Tissue of origin | Sex | Age (years) |
|---|---|---|---|
| LJ-136 | Pleural effusion in a patient with breast carcinoma | F | 60 |
| PD-201 | Solid tumor, carcinoma of colon | M | 58 |
| NB-208 | Solid tumor, invasive carcinoma of bladder | M | 69 |
| LC-207 | Metastasis of lung carcinoma to supraclavicular lymph node | M | 74 |
| JM-164 | Peritoneal effusion, associated with carcinoma of ovary | F | 57 |
| DUR | Pleural effusion in a patient with lung carcinoma | M | 63 |
| PA | Peritoneal effusion, carcinomatosis | F | 87 |
| EB-216 | Solid tumor, left lung, alveolar cell carcinoma | M | 67 |
| LY-190 | Solid tumor, endometrial carcinoma | F | 61 |
| GB | Peritoneal effusion, extensive abdominal carcinomatosis. Primary cancer (probably stomach) | M | 45 |
| EA-225 | Solid tumor, right lung, squamous carcinoma | M | 73 |

*Bone marrow from an untreated case of chronic granulocytic leukemia*

One case of chronic granulocytic leukemia was studied. The chromosome preparations are from bone marrow, taken *before* any therapy was started. This case definitely shows the classical Philadelphia chromosome.

*Cell line from Burkitt's lymphoma*

A chromosome preparation was made from a suspension culture (Jijoye) of a cell line derived from a case of Burkitt's lymphoma. This apparently malignant disease is of special interest because of the high likelihood of its being of viral origin.

### *The Experimental Results—Human Cell Lines*

The Boveri concept hypothesizes a chromosomal imbalance that may be the *sine qua non* of malignancy. This could be an excess number of a specific class of chromosomes per cell, a deficiency in number of such chromosomes per cell, or an imbalance in the ratio of one class of chromosomes to some other class. If such a *sine qua non* exists for all malignancy, the same chromosomal imbalance should exist for all cell lines and all cancers. From our previously reported studies of 7 human cell lines plus 2 freshly obtained human cancers, E16 chromosomes were consistently elevated, expressed as average number of E16 chromosomes per cell. Even after correcting for any increase in *total* number of chromosomes per cell, both in cell lines and in fresh cancers, the E16 chromosome level was still significantly elevated in all cell lines and fresh cancers. Lastly, imbalances, expressed as the ratio of one class of chromosomes to another class, were consistently found only for ratios involving E16 chromosomes.

*Absolute chromosome levels in the present studies*

Data are now available for 17 human cell lines and for 11 freshly o human cancers, representing more than a three-fold increase in the total ı of entities studied. It is therefore possible to determine whether the e series still shows consistency with respect to the E16 elevation, as well, of to determine whether any additional regularities have appeared. The mental chromosome results on human diploid cells are presented in Tal The experimental results for the human cell lines are presented in Table I

TABLE III.—*The Chromosome Composition of Normal Diploid Cells**

(Mean = mean number of chromosomes cell; SE = standard error of mean)

| | Normal Diploid Males (Blood Lymphocytes + Tissue Fibroblasts) 872 metaphases from 11 subjects 46·00 chromosomes/cell | | Normal Diploid Females (Blood Lymphocytes + Tissue Fibroblasts) 962 metaphases from 17 subjects 46·00 chromosomes/cell | |
|---|---|---|---|---|
| Chromosome class | Mean | ±SE | Mean | ±SE |
| A1 | 2·00 | 0·016 | 2·02 | 0·018 |
| A2 | 2·03 | 0·017 | 2·00 | 0·019 |
| A3 | 1·99 | 0·022 | 2·01 | 0·023 |
| B | 4·04 | 0·028 | 3·98 | 0·027 |
| C+X | 14·91 | 0·034 | 15·95 | 0·034 |
| D | 5·96 | 0·010 | 5·98 | 0·007 |
| E16 | 2·06 | 0·031 | 2·05 | 0·031 |
| E (17–18) | 3·97 | 0·030 | 3·93 | 0·031 |
| F | 3·99 | 0·026 | 4·03 | 0·023 |
| G+Y | 5·01 | 0·009 | 4·01 | 0·005 |
| Marker 1 | | | | |
| Marker 2 | | | | |
| Marker 3 | 0·01 | 0·003 | 0·01 | 0·003 |
| Marker 4 | | | | |
| Marker 5 | 0·01 | 0·004 | 0·02 | 0·004 |
| Marker 6 | | | | |
| Marker 8 | | | | |
| Marker 9 | | | | |
| Marker 12 | | | | |
| Marker 13 | | | | |
| Marker 14 | | | | |
| Marker 15 | 0·02 | 0·005 | 0·01 | 0·004 |

* The values for all classes are almost in perfect agreement with Denver classification, i the satisfactory character of the cutting lines of Fig. 1(a).

As was anticipated over 60 years ago by Boveri, numerous highly sig elevations and depressions in mean number of specific chromosome clas found for the various cell lines studied.* But isolated elevations or dep are of no real interest in evaluation of the Boveri hypothesis. If a pa chromosome imbalance is to be a *sine qua non* of malignant behavior, t balance must be present in all the cell lines and cancers studied. T question we must ask, for the human cell lines, is " Are there any chror elevations (or depressions) in mean number per cell that are found in all lines? " The answer is that only for the E16 chromosome class can co behavior be found for all the cell lines presented here. No other chror class is significantly elevated or significantly depressed in all the cell lines mean chromosome number per cell is elevated in every cell line studied.

* Detailed analyses for every chromosome class is available to the interested reader upo to the authors.

TABLE IV.—*Comparison of Mean E16 Chromosomes/Cell for all Human Cell Lines with Corresponding Normal Diploid Cells*

| Cell line | Number of metaphases measured | Mean number of chromosomes per cell | Mean number of E16 chromosomes/cell: E16 in cell line | E16 in normals | Difference | t Value† | Ratio (E16 in cell line / E16 in normals) |
|---|---|---|---|---|---|---|---|
| HeLa | 43 | 69·72 | 8·23 | 2·05 | 6·18 | 20·67 | 4·02 |
| Detroit-6 | 40 | 64·67 | 5·63 | 2·06 | 3·57 | 15·58 | 2·74 |
| KB | 41 | 68·78 | 5·66 | 2·06 | 3·60 | 11·65 | 2·75 |
| HEp-2 | 37 | 75·35 | 8·22 | 2·06 | 6·16 | 16·68 | 4·00 |
| J-111 | 28 | 111·82 | 7·75 | 2·05 | 5·70 | 13·14 | 3·78 |
| RPMI-2650* | 136 | 46·00 | 2·79 | 2·06 | 0·73 | 10·79 | 1·36 |
| Minnesota-EE | 42 | 68·21 | 6·10 | 2·06 | 4·04 | 14·55 | 2·97 |
| Intestine-407 | 37 | 77·32 | 8·04 | 2·05 | 5·99 | 17·32 | 3·92 |
| Chang Liver | 45 | 68·36 | 7·78 | 2·05 | 5·73 | 21·14 | 3·79 |
| Detroit-98 | 132 | 63·66 | 5·45 | 2·06 | 3·39 | 27·48 | 2·65 |
| AV-3 | 50 | 76·18 | 7·07 | 2·05 | 5·02 | 22·09 | 3·45 |
| WISH | 52 | 72·88 | 4·73 | 2·05 | 2·68 | 12·73 | 2·31 |
| Girardi Heart | 46 | 80·00 | 5·13 | 2·06 | 3·08 | 14·94 | 2·50 |
| FL | 46 | 62·74 | 5·90 | 2·05 | 3·85 | 16·30 | 2·88 |
| Detroit-98/AG | 148 | 63·76 | 7·16 | 2·06 | 5·10 | 37·52 | 3·48 |
| Detroit-98/AH-2 | 51 | 62·06 | 7·06 | 2·06 | 5·00 | 19·52 | 3·44 |
| Detroit-98/AH-R | 50 | 62·66 | 6·88 | 2·06 | 4·82 | 21·17 | 3·35 |
| Mean for all cell lines | | | 6·36 | 2·05 | 4·31 | | 3·10 |

* The only human cell line with 46 chromosomes per cell.

† For a *t* value ≃3·0, the significance level is 1%. For the very high *t* values in this table, the results are significant far beyond the 1% level.

The elevation in mean number of E16 chromosomes per cell is highly significant in every case (for the *t* values in Table IV, all *P* values are <0·0001). Further, the elevation in E16 level is marked, averaging 3·10-fold for the entire group. The only moderate elevation is in RPMI-2650, where a 36% elevation in E16 level is noted.

*Chromosome levels corrected for total number of chromosomes per cell*

Inspection of Table IV shows that almost every cell line shows a mean total number of chromosomes greater than the 46 chromosomes characteristic of normal cells. The only exceptional cell line is RPMI-2650 which does show 46 total chromosomes, but with an abnormal distribution of chromosomes including E16 elevation. The question can be raised whether the observed E16 elevations in the other cell lines is simply a reflection of the increase in total number of chromosomes per cell. This is definitely not the case. First, if such were the explanation, it is odd that no chromosome class other than E16 shows consistent elevation in all the cell lines. Second, by direct test it is readily demonstrable that the E16 elevation is significantly and appreciably greater than that expected from the elevation in total chromosome number per cell.

We shall henceforth refer to the number of chromosomes expected after correcting for total number of chromosomes per cell as the " corrected expectation ".

Such analysis has been carried through for every chromosome class in every cell line studied. Again, only for E16 chromosomes is consistent behavior observed-namely, an elevation of E16 mean chromosome number above the " corrected expectation ". The mean E16 level is 2·03 times the "corrected E16 expectation ".

Gartler (1968) has raised the question of whether several of the cell lines American Type Culture Collection might not be independent, but were p derived by culture contamination with HeLa cells. His evidence is basec studies of isozymes of glucose-6-phosphate dehydrogenase and phosphc mutase. For argument sake, let us assume this may be true. If true indeed of great interest that there are such gross chromosomal comp differences, both in total number and distribution into the various classes noted in Table IV but with all cell lines showing marked E16 chromosome It can be argued, therefore, that whether the cell lines originated indepen or whether some may have derived from HeLa cells, they can evolve to di chromosome composition, but that if they are to become " immortal " cel capable of producing cancer on heterotransplantation, they must possess tl chromosome excess. Thus, we do not believe Gartler's concern over origin cell lines would alter the importance of the demonstrated E16 excess in ] cell lines. And, of course, Gartler's evidence is totally irrelevant for the st confirmatory evidence derived from the E16 excess in the 11 fresh cancers, HeLa contamination is out of the question.

*Experimental results—freshly obtained human cancers*

All the data presented to this point confirm our previous hypothesis t E16 chromosome level elevation, absolute or relative to other chromosome c characterizes human cell lines—cell lines regarded by many as malignan *vitro*. How well does this hypothesis hold up in freshly obtained human ca This latter represents the ultimately desirable test material, for with such m the argument cannot be raised that long-continued cell culture accounts 1 chromosome findings, rather than a relationship of chromosome constitutio malignancy *per se*. Eleven fresh cancers have now been studied, in ever with chromosome preparations made between 2–20 hours after excision malignant tissue or withdrawal of the malignant effusion. The nature material was described in Table II. The E16 data for all the 11 fresh canc presented in Table V.

TABLE V.—*E16 Chromosome Levels in* 11 *Freshly Obtained Human Can*

| Cancer case | Sex | No. of meta-phases studied | Mean total No. of chromo-somes/cell | E16 in cancers chromo-somes/cell | E16 in normals chromo-somes/cell | Difference | Ratio (E16 Cancer / E16 Control) |
|---|---|---|---|---|---|---|---|
| LJ-136 | F | 116 | 81·41 | 4·49 | 2·05 | 2·44 | 2·19 |
| PD-201 | M | 25 | 73·56 | 6·04 | 2·06 | 3·99 | 2·94 |
| NB-208 | M | 75 | 48·85 | 3·41 | 2·06 | 1·36 | 1·66 |
| LC-207 | M | 26 | 66·15 | 4·31 | 2·06 | 2·25 | 2·10 |
| JM-164 | F | 23 | 74·43 | 4·61 | 2·05 | 2·56 | 2·25 |
| DUR | M | 33 | 68·15 | 3·97 | 2·06 | 1·92 | 1·93 |
| PA | F | 50 | 47·40 | 2·96 | 2·05 | 0·91 | 1·45 |
| EB-216 | M | 26 | 69·35 | 3·19 | 2·06 | 1·14 | 1·55 |
| LY-190 | F | 13 | 47·00 | 3·23 | 2·05 | 1·18 | 1·58 |
| GB | M | 76 | 48·24 | 2·46 | 2·06 | 0·40 | 1·20 |
| EA-225 | M | 53 | 37·13 | 2·11 | 2·06 | 0·05 | 1·03 |
| Mean | | | | 3·72 | 2·05 | 1·66 | 1·81 |

*Note:* Both in solid tumor tissues and malignant effusions, cells are commonly encounte 46 chromosomes in addition to those possessing an abnormal number of chromosomes. ' with 46 chromosomes are *not* analyzed, since it is impossible to know whether they are can normal stromal cells, or a mixture of both. Only cells with a *total* number of chromosomes a the abnormal mode are reported.

Ten of the 11 fresh cancers show appreciable and highly significant elevations in absolute E16 chromosome level. One case, EA-225, cannot be proved to show an absolute elevation in E16 level. This one cancer is extremely unusual in that it has only 37·13 total chromosomes per cell. Such cancers with total chromosome numbers this low have been reported before, but they are extremely rare. As will be noted below, when the E16 level is considered relative to the total number of chromosomes, the elevation in E16 level compared with the " corrected expectation " for 37 total chromosomes *is* highly significant.

Analysis made of every other chromosome class demonstrate, for the 11 fresh cancers, as for the 17 cell lines, no chromosome class other than E16 shows consistent elevation—nor does any other class show consistency in the form of a depressed chromosome level per cell. These analyses are available upon request.

*E16 chromosome levels corrected for total number of chromosomes per cell*

The " corrected expectation " for any class of chromosomes is the number of chromosomes of that class expected after correcting for total number of chromosomes per cell. Ten of the 11 fresh cancers show significant E16 chromosome elevation above the "corrected expectation." Case EA-225, with the very low total of 37 chromosomes per cell meets this criterion. Case EB-216, with a demonstrable E16 level elevation on an absolute basis fails to meet the more rigorous criterion of elevation above " corrected expectation ".

*E-16 levels in SV-40 virus altered human cells*

Shein and Enders (1962) first demonstrated that the virus of Simian origin, SV-40, can regularly accomplish the alteration of normal human cells to immortal malignant-behaving cell lines. This same SV-40 virus has been proved definitively to produce cancer in two species, including newborn hamsters and *Mastomys*.

In the " materials " section above one human cell line was described which was produced *in vitro* by the action of SV-40 viruses upon normal human cells in culture. The chromosome analysis for this cell line, together with comparisons with chromosome levels in normal controls is presented in Table VI. It is clear from these data that the E16 chromosome level is markedly elevated in the cell line produced by SV-40 alteration of normal cells, approximately 2·39 times the normal cell E16 content. Since the total number of chromosomes in the SV-40 altered cells is 81·73 chromosomes per cell, the " corrected expectation " for E16 chromosomes is $[(81{\cdot}73/46{\cdot}00) \times 2{\cdot}05] = 3{\cdot}64$ per cell. But the observed E16 chromosome content is 4·90 chromosomes per cell. The difference, 1·26 chromosomes per cell, is still large, and the probability of this not being real, with a $t$ value $= 6{\cdot}71$ is $P < 0{\cdot}0001$. Thus, not only is the E16 chromosome level absolutely elevated, but also it is elevated markedly even after correcting for total number of chromosomes per cell.

Further inspection of the data of Table VI shows that more marker classes of chromosomes are represented at significant occurrence frequencies than for any other human cell line or human cancer presented earlier in this communication. Since marker chromosomes are presumed to arise by injuries to normal chromosome classes, the inference is that the SV-40 virus has produced extensive chromosomal injury during the process of alteration of normal cells to a malignant cell line. That such is the case is evident from careful study of many metaphases not

TABLE VI.—*The Chromosome Constitution of a Human Cell Line Prod[illegible] SV-40 Virus—Contrast with Normal Human Diploid Female Cells*

77 metaphases of SV-40 line studied. 81·73 chromosomes/cell

| Chromosome class | SV-40 line | Normal controls | Difference | *t* test | Ratio (Chromosome/cell in / Chromosome/cell in) |
|---|---|---|---|---|---|
| A1 | 1·92±0·11 | 2·02±0·02 | −0·10 | 0·90 | 0·95 |
| A2 | 4·12±0·15 | 2·00±0·02 | 2·12 | 13·74 | 2·05 |
| A3 | 4·71±0·17 | 2·01±0·02 | 2·70 | 16·04 | 2·34 |
| B | 7·47±0·19 | 3·98±0·03 | 3·49 | 18·50 | 1·88 |
| C+X | 21·13±0·34 | 15·95±0·03 | 5·18 | 15·12 | 1·33 |
| D | 10·34±0·21 | 5·98±0·01 | 4·36 | 20·39 | 1·73 |
| E16 | 4·90±0·18 | 2·05±0·03 | 2·85 | 15·67 | 2·39 |
| E(17–18) | 7·78±0·25 | 3·93±0·03 | 3·85 | 15·58 | 1·98 |
| F | 4·00±0·17 | 4·03±0·02 | −0·03 | −0·18 | 0·99 |
| G+Y. | 9·08±0·24 | 4·01±0·01 | 5·07 | 21·49 | 2·27 |
| Marker 1 | 1·46±0·12 | 0·00 | 1·46 | 11·91 | —* |
| Marker 3 | 1·46±0·11 | 0·01±0·003 | 1·45 | 13·14 | — |
| Marker 4 | 0·62±0·08 | 0·00 | 0·62 | 7·63 | — |
| Marker 5 | 0·25±0·09 | 0·02±0·004 | 0·23 | 2·52 | — |
| Marker 6 | 0·09±0·03 | 0·00 | 0·09 | 2·72 | — |
| Marker 8 | 0·03±0·02 | 0·00 | 0·03 | 1·41 | — |
| Marker 9 | 0·05±0·03 | 0·00 | 0·05 | 2·01 | — |
| Marker 11 | 0·57±0·10 | 0·00 | 0·57 | 5·83 | — |
| Marker 12 | 0·01±0·01 | 0·00 | 0·01 | 0·99 | — |
| Marker 13 | 0·03±0·02 | 0·00 | 0·03 | 1·41 | — |
| Marker 14 | 0·77±0·08 | 0·00 | 0·77 | 10·26 | — |
| Marker 15 | 0·96±0·12 | 0·01±0·004 | 0·95 | 8·16 | — |

* The extremely low or zero values for the marker classes in normal control material ren[illegible] involving such classes of dubious meaning. Hence such ratios are not recorded. The however, for the marker classes, can be relied upon to evaluate the probabilities that the marker chromosome contents are real.

included in Table VI. Chromosome pulverization, chromosome distortion [illegible] recognition, and other anomalous chromosome distortions are noted re[illegible] many metaphases unsuitable for analysis. The true chromosomal alt[illegible] *in toto* is even more extensive, therefore, than presented in Table VI. As [illegible] of the extensive representation of markers, it would be particularly hazar[illegible] assign much meaning to ratios of one chromosome class to another in these virus-altered cells.

### *Possible Exceptions (?) to the E16 Hypothesis*

### *Chronic granulocytic leukemia*

Nowell and Hungerford's (1960) discovery of the Philadelphia chromos[illegible] chronic granulocytic leukemia was the first specific chromosome abnorm[illegible] a malignant disease. This chromosome is considered to represent a G chron[illegible] from which approximately 40% of the DNA content has been deleted. Th[illegible] no indication reported of an E16 chromosome excess. We have now [illegible] opportunity to examine a case of chronic granulocytic leukemia by quant[illegible] chromosome analysis. No excess of E-16 chromosomes was demonstrable

The deletion of some G chromosomal material means that the G + Y lower than normal in chronic granulocytic leukemia. Therefore, even normal absolute E16 level, the ratio E16/G + Y may well be higher, in effec[illegible] normal. It is to be noted that this ratio, E16/G + Y, is high in every hum[illegible] line and every one of the 11 fresh cancers studied. It is, therefore, possib[illegible]

chronic granulocytic leukemia may not represent an exception to the E16 hypothesis. Final decision must await determination of whether the E16/G + Y imbalance is a necessary or sufficient condition for malignancy.

*Burkitt's lymphoma*

Burkitt's lymphoma is a special case among malignancies in two major respects. First, virus or virus-like particles are commonly demonstrable in involved tissues. Second, cell lines drived from Burkitt's lymphoma grow only in suspension culture, in contrast to those described above, which all grow as monolayers, but which may also grow in suspension culture. One such cell line (JIJOYE) has been subjected to quantitative chromosome analysis. The E16 level was within normal limits both in the Burkitt's lymphoma cells with 46 and those with 47 chromosomes. Every cell, however, shows a specific marker chromosome (Marker 12, Fig. 1(b)), the genetic content of which is unknown. If E16 genetic material is present in this marker chromosome, Burkitt's lymphoma would be consistent with all the cell lines and cancers. If not, this will represent a distinct exception to the E16 hypothesis.

*Corroborative evidence from the literature*

In a problem of the magnitude of evaluation of the E16 hypothesis it is clear that no one laboratory is ultimately going to be able to provide the breadth of observations required. Unfortunately, in the semi-quantitative state of chromosome studies broadly, there are very few reports of chromosomes in malignancy where quantitative characterization of chromosomes is provided. In general the reports are of the " cut-out " karyotype variety for one or a few cells per case. In the absence of quantitative parameters it is commonly found that chromosome assignments are equivocal. Regardless of their reported interpretation, we feel there are several suggestive and some straightforward corroborative examples presented in recent literature (Messinetti *et al.*, 1968; Ayraud and Kermarec, 1968; McAllister *et al.*, 1969; Crossen *et al.*, 1969; Rigby, 1968, Ponten and Saksela, 1967).

Our sincere thanks to the many people who contributed in a variety of indispensable ways to this research effort. Dolores Piluso, Margaret Soderberg and Erma Kovich provided much help in the laborious process of tracing metaphases. Frank Fickel designed, executed, and maintained our microprojection hardware with technical and photographic assistance from David Dixon and Richard Krumhansl of the Technical Photography Group at LRL, Livermore. Stuart Stone provided the sophisticated character recognition programs required for computer measurements and calculations, with the help of A. Landon Bruce. James L. Littlepage provided electronic scanning of the tracing photographs. Charlie Pierce gave us excellent service as a reference librarian. And special thanks to Lillian Rachlin, M.D., and her staff of the Department of Surgery, U.S. Veterans' Hospital, Livermore, California, for supplying us with large numbers of fresh cancer specimens.

This work was supported by the U.S. Atomic Energy Commission.

### REFERENCES

AMERICAN TYPE CULTURE COLLECTION—(1967) ' Registry of Animal Cell Lines Certified by the Cell Culture Collection Committee ', 2nd Supplement. Rockville, Maryland (American Type Culture Collection Cell Repository).

AYRAUD, N. AND KERMAREC, J.—(1968) *Bull. Ass. fr. Étude Cancer*, **55**, 91.

BOVERI, T. (?) *Verh. phys.-med. Ges. Wurzb.*, Bd 35.—(1929) ' The Origin of Malignant Tumors ', translated by M. Boveri. Baltimore (Williams and Wilkins).

CROSSEN, P. E., FITZGERALD, P. H., MENZIES, R. C. AND BRETHAUT, L. A.—(1969) *J. med. Genet.*, **6**, 95.

GARTLER, S. M.—(1968) *Nature, Lond.*, **217**, 750.

GOFMAN, J., MINKLER, J. AND TANDY, R.—(1967) ' A Specific Common Chromosomal Pathway for the Origin of Human Malignancy '. University of California Lawrence Radiation Laboratory Reports (UCRL-50356), November 20.

HANDLER, A. H. AND FOLEY, G. E.—(1956) *Proc. Soc. exp. Biol. Med.*, **91**, 237.

HAYFLICK, L.—(1965) *Expl Cell Res.*, **37**, 614.

MCALLISTER, R. M., MELNYK, J., FINKELSTEIN, J. Z., ADAMS, E. C., JR. AND GARDNER, M. B.—(1969) *Cancer, N.Y.*, **24**, 520.

MESSINETTI, S., ZELLI, G. P., MARCELLINO, L. R. AND ALCINI, E.—(1968) *Cancer, N.Y.*, **21**, 1000.

NOWELL, P. C. AND HUNGERFORD, D. A.—(1960) *J. natn. Cancer Inst.*, **25**, 85.

PONTEN, J. AND SAKSELA, E.—(1967) *Int. J. Cancer*, **2**, 434.

RIGBY, E.—(1968) *Br. J. Cancer*, **22**, 480.

SHEIN, H. M. AND ENDERS, J. F.—(1962) *Proc. natn. Acad. Sci. U.S.A.*, **48**, 1164.

SOUTHAM, C. M., MOORE, A. E. AND RHOADS, C. P.—(1957) *Science, N.Y.*, **125**, 158.

STONE, S. P.—(1967) ' Chromosomal Scanning Program at LRL. Part I. A Set of Chromosome Pattern-Recognition Programs '. University of California Lawrence Radiation Laboratory Reports (UCRL-50364 Part I), November 25.

STONE, S. P. AND LITTLEPAGE, J. L.—(1967) ' The Chromosome Scanning Program at Lawrence Radiation Laboratory '. (UCRL-70413).

STONE, S. P., LITTLEPAGE, J. L. AND CLEGG, B. R.—(1969) ' Second Report on the Chromosome Scanning Program at the Lawrence Radiation Laboratory ' (UCRL-71493).

ZANG, K. D. AND SINGER, H.—(1968) *Angew. Chem.* (International Edition), **7**, 709, 718.